# Bioremediation of polluted soil due to tsunami by using recycled waste glass

**DOI:** 10.1038/s41598-021-93806-4

**Published:** 2021-07-12

**Authors:** M. Azizul Moqsud

**Affiliations:** Department of Civil and Environmental Engineering, Yamaguchi University, Ube City, 755-8611 Japan

**Keywords:** Environmental sciences, Environmental chemistry

## Abstract

In this research, bioremediation of tsunami-affected polluted soil has been conducted by using collective microorganisms and recycled waste glass. The Tohoku earthquake, which was a mega earthquake in Japan triggered a huge tsunami on March 11th, 2011 that caused immeasurable damage to the geo-environmental conditions by polluting the soil with heavy metals and excessive salt content. Traditional methods to clean this polluted soil was not possible due to the excess cost and efforts. Laboratory experiments were conducted to examine the capability of bioremediation of saline soil by using recycled waste glass. Different collective microorganisms which were incubated inside the laboratory were used. The electrical conductivity (EC) was measured at different specified depths. It was noticed that the electrical conductivity decreased with the assist of the microbial metabolisms significantly. Collective microorganisms (CM2) were the highly capable to reduce salinity (up to 75%) while using recycled waste glass as their habitat.

## Introduction

The Great East Japan Earthquake was one of the largest earthquakes (M.9.0) in the world, which caused a huge-scale tsunami in the northern part of Japan on 11th March 2011. The tsunami caused significant damage to geotechnical and geo-environmental engineering perspective^1^. According to the Geospatial Information Authority of Japan, 561 km^2^ was flooded by the seawater: 24 km^2^ in Aomori, 58 km^2^ in Iwate, 327 km^2^ in Miyagi, 112 km^2^ in Fukushima, 23 km^2^ in Ibaraki, and 17 km^2^ in Chiba, which are in the northern part of Japan. This earthquake and consequent tsunami ensued in a disaster, with huge damage of human life and vast destruction to buildings and infrastructure. Salinity of agricultural land is one of the most serious geo-environmental problems caused by tsunamis. Salinity is also a major geo-environmental problem in the various parts of the world as most of the principal crops cannot grow and consequently the land becomes a desert in saline soil areas. Saline soil is a main limiting production issue globally for every major crop including rice, wheat, corn, and vegetables^2–4^. Although the tsunami is the main reason for the salinity of the soil, however, when rainfall is inadequate to leach ions from the soil profile, salts collect in the soil, resulting in soil salinity^5,6^. In arid areas, the soils frequently become saline with very low agricultural potential and various crops are grown under irrigation. However, insufficient irrigation management leads to secondary salinization that involves 20% of irrigated lands globally^7–9^. The recent trend of climate change and sea level rise increases the challenges of salt concentration near coastal areas.

The conventional way of reducing salt from soil is impossible due to the shortage of fresh water and the method of leaching in the tsunami affected areas. To deal with this dilemma, vegetative and microbial bioremediation of saline soils has become apparent as an encouraging procedure. Farming of inexpensively helpful halophytes, salt-tolerant plants, and crop mixtures capable of growing under salt-stress conditions has facilitated transfer of saline and sodic wastelands^8,9^. The removal of salinity was tried with halo bacteria and compost in the previous studies by Moqsud and Omine (2013)^8,10^. Nevertheless, this process needs to grow halo bacteria and to produce compost are not easy and also time consuming. Therefore, bioremediation of saline soil is essential with some easily accessible common microbes. Many researchers are trying to overcome this geoenvironmental engineering problem in recent years. Wu *et al.* (2016) shows an innovative approach that couples electrokinetics with microbial degradation to breakdown hydrocarbons in polluted saline soil^10,11^. However, the approach was expensive and cannot use for the vast areas. There are efforts to restore saline soil and to plant some medicinal plants in the arid areas^12^. However, due to the higher salt content, plants cannot grow properly.

Resource recovery from glass waste is a great concern both in the developing countries and the industrial parts of the world. Though the recycled waste glass named as foamed waste glass (FWG) is a commercially available geo-materials in Japan, it had not been used widely as a matter of bioremediation of polluted soil or the potential habitat of the microorganisms in the past. The recycled waste glass has lot of pore spaces. These pore spaces can be the habitat of the microbes in the clay soil. The native soil such as rice field soil is mainly clay and can provide very small amount of oxygen to the aerobic bacteria under the wet condition. For this reason, the porous foamed waste glass (recycled waste glass) had been used in this research as a potential habitat to live and grow with the trapped oxygen inside the porous recycled waste glass during the bioremediation process.

The main intention of this research is therefore to monitor the bioremediation of saline soils through some common collective microbes in the laboratory which can be applied in the tsunami affected saline soil in the field. The other objective of this research is to use the recycled waste glass (FWG) in the bioremediation of saline soil as the potential habitat of the living microorganisms.

## Results

**Figure 1:**
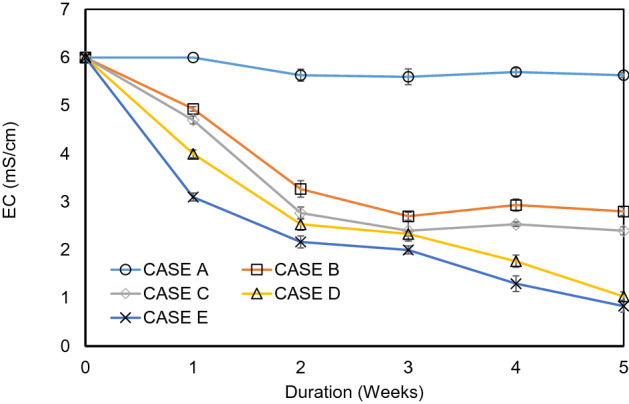
Variation of Electrical Conductivity (EC) with duration in experiment 1.

Figure 1 demonstrates the result of 1st experiment which shows the variation of EC with duration. Case A was a control sample and did not apply any additional microorganisms. The EC of Case A did not decrease throughout the study period (5 weeks). The EC of the other cases reduced with duration due to bioremediation of the saline soil. The EC lowered successfully when microbial mixture CM 1 and CM2 were used instead of having one specific bacteria (case B, C). In the case of E (CM 2), the EC value reduced significantly after 5 weeks.

**Figure 2:**
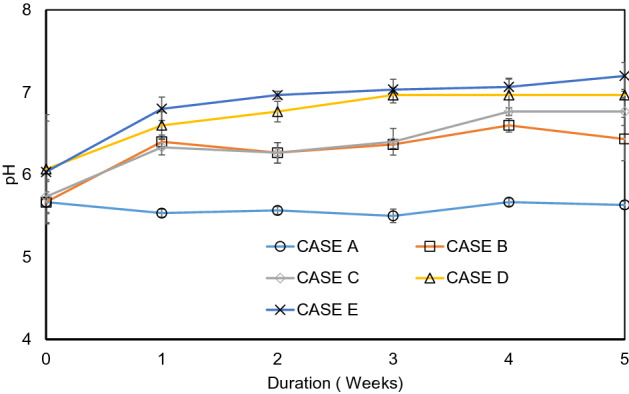
Variation of pH with duration in experiment 1.

Figure 2 illustrates the variation of pH with duration in experiment 1. pH value increased gradually and reached at a certain level during the experiment period. For the case of E using CM2, the pH value reached at the most favorable range of near 7, which indicates that the collective microorganism CM 2 is not only lowered the salt content but also improved the geo-environment by changing the acidic condition of the soil. The *photosynthetic bacteria* in CM2 had a bigger influence to decrease the salinity. Zhang *et al.* (2020) shows that pH is a great indicator of the remediation of soil^29^. In this study, no significant changes (p ≺ 0.05) in the soil pH were observed in blank sample throughout the experiment. The result was similar to some previous studies^13,14^. The soil pH is the most critical parameter in the EK-Bio process also^15,16^. Soil pH values below three and above nine, as well as sudden changes in the pH of the remediate soil, can significantly inhibit microbial growth^17^. The pH of the bioremediation of saline soil was not significantly changed during and after 3 weeks of the experiment which indicated a safe and effective remediation of the excess salt content from the soil.

**Figure 3:**
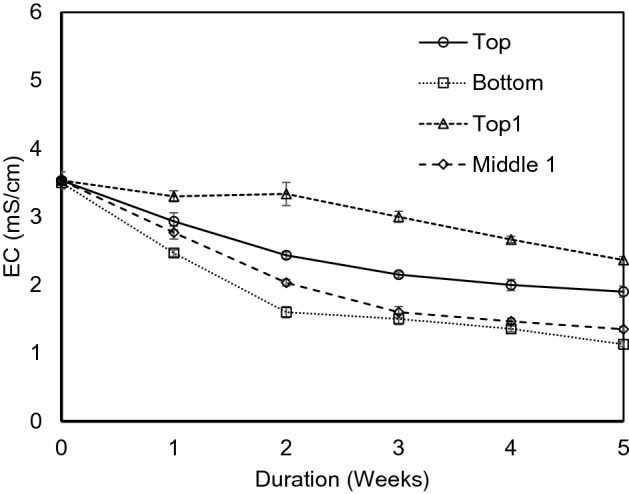
Variation of Electrical Conductivity (EC) with duration in experiment 2.

Figure 3 demonstrates that EC decreased when FWG was used as a layer both in middle and at the bottom in experiment 2. EC lowered most near the FWG layers. Bottom and top1 and middle 1 showed higher decline of EC than the other spots of the sample collection for EC measurement. The saline soils are controlled by many types of halophilic and halotolerant microorganisms, spread over many phylogenic groups. The halophilic microbes have potential for bioremediation of salt-dominant soils. By applying the porous FWG layer in the soil environment, the halophilic bacteria find some suitable place for their inhabitants and become more active gradually. The higher number of the microorganisms can increase the activities and consequently increase the reduction of salinity from the tsunami affected soil in the field. It was discussed in the field investigation section that; a lot of tsunami debris were mixed, and the tsunami deposits were altered the basic soil properties of the agriculture field. So, the use of FWG can improve the situation of the geo-environmental condition and improve the habitat of the effective microorganisms.

**Figure 4:**
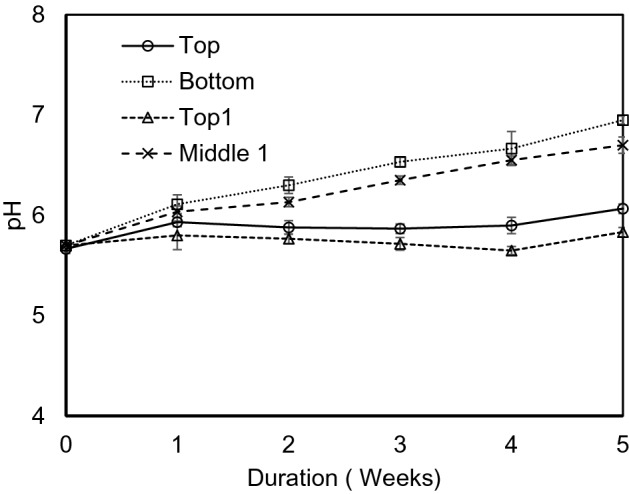
Variation of pH with duration in experiment 2.

Figure 4 demonstrates that pH values varied with duration in experiment 2. The trend indicates that pH rose slowly. The maximum pH value was observed at the bottom and middle 1 sampling points. The pH value primarily improved after leaching of the soil and the rate of salt leaching influenced the soil pH value. This is caused by washing out of chloride ion and sulfate ion relative to bicarbonate ion. Nevertheless, as the water content was very minimal and no water was leached for the duration of the experimentation, the increase of pH value is owing to the activities of microorganisms during the bioremediation process. The pH value was in increasing trend but remained in the suitable range for geo-environment throughout the experiment.

**Figure 5:**
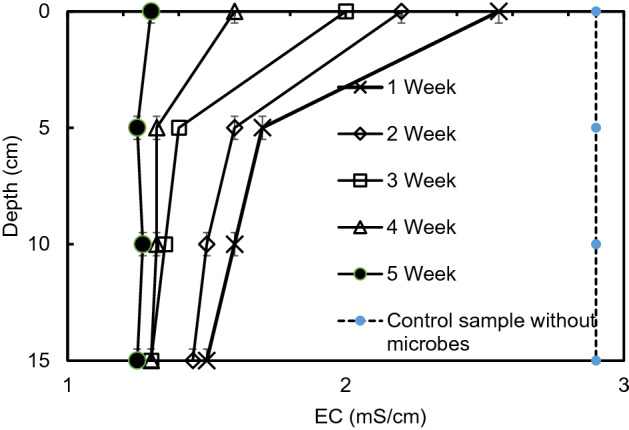
Variation of EC with duration in experiment 3 in different depths.

Figure 5 indicates the difference of EC with time at various depths in laboratory test 3. The EC progressively decreased with duration. The decrease of EC value was greater at the lower depth than at the surface areas. Saline water evaporates and the salt collected near the surface, and this is the probable reason for the high salt content near the surface of the saline soil^18–20^. After the 5th week, the EC decreased in all the depths measured. When FWG was mixed (in Experiment 3), the bioremediation spreads throughout the saline soil. The pore spaces of FWG gave some inhabitable places for the microorganisms used in the bioremediation method. The EC decreased almost 75% within 5 weeks by using the effective microorganisms (CM2) in the experiment. This type of EC is appropriate for the development of plants in the soil. However, when the control sample without microbes was used then the EC value did not change significantly over time which indicates the microbes play the vital role in the experiment 3 rather than FWG.

**Figure 6:**
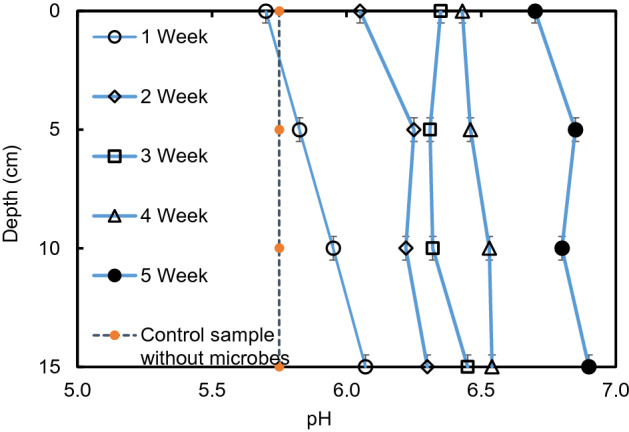
Variation of pH with duration in experiment 3 in different depths.

Figure 6 explains the variation of pH with time at various depths. The pH values increased gradually with time. The microorganisms work altered the soil chemistry and produced more alkaline condition. The pH value of FWG is higher (8.1) than the soil used (5.7) during the initial state of the experiment. At the end of the experiment (5th week), the pH value reached at nearly 7 in all the depths. This indicates that the bioremediation with FWG could be effective in the salinity removal of the saline soil which is in acidic condition initially. A control sample without microorganisms was also conducted to prove the theory and found that the control sample did not show any changes, and the chemical properties of the soil were not affected by the recycled waste glass^21,22^.

## Discussion

**Figure 7:**
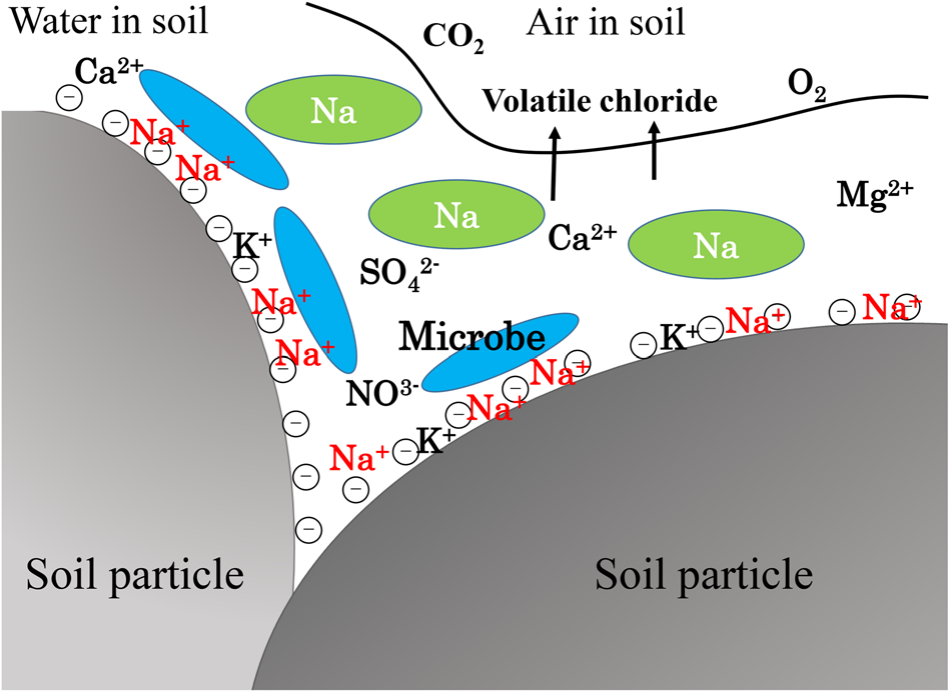
Schematic diagram of the mechanism of bioremediation of saline soil.

The reduction of electrical conductivity was about 75% of the initial salinity due to the symbiotic action among the microorganisms^23,24^. Daghio *et al.* (2018) explains the bio-electrochemical BTEX removal at different voltages and found that the variation of microbial communities is normal during the process of the bioremediation^21^. Wu *et al.* (2019) shows an application of molecular biological tools for monitoring efficiency of trichloroethylene remediation^10,11^. Nevertheless, the salinity problem is not hazardous in terms of carcinogens and the symbiotic activities can be a main reason for the reduction of salt content during the process. The overall efficient amount of electrical conductivity was reduced when the recycled waste glass was mixed thoroughly with the saline soil with the collective microorganisms. This process indicates that the salinity of the soil can be reduced significantly by using the recycled waste glass. The potential mechanism of the bioremediation by using collective microorganisms are involved in the two different phenomena. The sodium chloride is ionized in the soil and became sodium ion and chloride ion. Then this ion can be reacted with the metabolic activities of the collective microorganisms. With the help of the microbial metabolic activities the sodium ion chelates and make new compound. The chloride ion has converted to gas and due to the bio-volatilization process escapes to the environment. Figure 7 shows the potential mechanism of the bioremediation of the saline soil with the collective microorganisms. However, the mechanism of gas released to the atmosphere should be studied more in the future experiment by using the gas chromatography.

## Conclusions

The saline soil can be treated with bioremediation by using the commonly available collective microorganisms. The efficiency of the bioremediation can be enhanced by the using of FWG as their habitat. Wang *et al.* (2020) describes that microbial electrochemistry of the bioremediation for the different polluted soil by using several microbes^16^. The collective microorganisms CM2 were more efficient than the specific microbes. The reason of this trend is that, when several microbes are mixed, they work together and support each other to grow and help to become active in the bioremediation process in a symbiosis process^25^. The recycled waste glass (FWG) helped to reduce the salinity through bioremediation in the tsunami affected soil in the laboratory experiments. This is anticipated to that point that FWG can be used as good place to live for the bacteria and increase their numbers inside the pores as a potential habitat. EC values reduced around 70–75% at the bottom areas when the collective microorganisms were applied in the bioremediation. One of the most important indicators of suitable geoenvironment for the bioremediation is pH and it was found in the favorable range throughout the bioremediation process (7–9). However, the field application of recycled waste glass (FWG) needs more research before applying in the agricultural field.

## Materials and methods

### Field investigation

Field investigation has been carried out in the tsunami affected areas. Soil samples were collected from the agricultural fields in different designated depths (0,5,10,20 cm). The basic properties of those soil samples, the electrical conductivity (EC) and the pH were checked as a monitoring of the geo-environmental condition. It was found that the salinity of the soil samples was higher than the recommended limit (0.6 mS/cm) for crop production due to tsunami water inundation throughout the depths. Though the pH value was within the safe limit (7–9) for all the cases the EC value was higher than the safe limit. The soil sample was also affected with the tsunami deposit and other tsunami debris. The maximum grain size of the samples was limited to smaller than 2 mm, and accordingly, all the deposits can be classified into sand or fine-grained soil. Another important finding was that the wet samples that were collected at the sites submerged for extended periods contained a considerably greater fine fraction, especially of particles finer than 10 μm. This is probably for the reason that the longer period of flood increased the sedimentation of the fines fractions^1^. Katsumi *et al.* (2011) examined some physical properties of 17 tsunami deposit samples collected at five tsunami-affected sites in early April 2011 in Miyagi Prefecture^26^. The water content of the tsunami sediments varied considerably. At some sites, the flood appeared for a prolonged period due to inadequate drainage system and the destruction of infrastructures. No organic chloride compounds were detected with concentrations higher than the environmental criteria for soil and groundwater quality even in the tsunami deposits areas^27^. This supports the claim that no extensive pollution occurred due to the earthquake and tsunami. However, in very limited spots, high concentrations of lead, n-hexane extracts and TPH (total petroleum hydrocarbon), which are an index of oil contamination, and PCB were discovered, even though there is no certain indication that the earthquake and tsunami triggered these pollutions (Inui *et al.*, 2012). Oguchi *et al.* (2012) characterized the chemical composition of tsunami deposits in combination with location of facilities dealing with toxic chemicals, and suggested that all tsunami deposit samples containing high level of hazardous chemicals were collected near the facilities where the particular detected chemicals were stored and/or used^24^.

### Laboratory experiments of bioremediation of saline soil with recycled waste glass (FWG)

#### Recycled waste glass

Resource recovery from waste material is a big problem all over the world and researchers are trying to use the recycled waste materials in various innovative ways^25,26^. In Japan, the recycling and resource recovery of broken glass had been a great challenge for a longer period. The recycled waste glass which is commercially available as foamed waste glass (FWG), was used in the experiments. FWG is used for embankment back-filling material in Japan as it is lightweight and durable. Due to its porous nature and the specific gravity, it was widely used as geo-materials. The specific gravity of the FWG used in the experiment was 1.774. With its porous structure, it could potentially hold some additional oxygen or water molecules for the living and growing of bacteria during the bioremediation process in the harsh environment such as  saline soil. FWG could be a good way to use as a geo-environmentally friendly material in the bioremediation process as a habitat of the microorganisms. In this study, the FWG is used as a material for the habitat of the collective microorganisms.

#### Laboratory test

**Figure 8:**
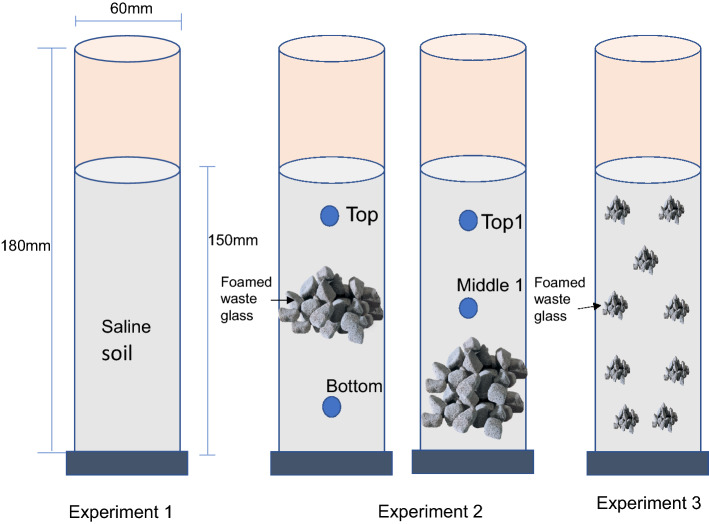
Schematic diagram of the column experiments for bioremediation of saline soil.

Three laboratory experiments (experiment1,2,3) were carried out to observe the effect of bioremediation on reducing the salinity in soil as shown in Fig. 8. In experiment 1 the different microbes were mixed with the saline soil for checking the effective microorganisms for salinity removal. In second experiment, the recycled waste glass was used as a layer in the middle and the bottom of the column to check the habitat with the collective microorganisms. In the third experiment, the FWG was mixed with the soil thoroughly. Artificial saline soil (collected from rice field of Yamaguchi University, Japan) was prepared in the laboratory by using artificial sea water^28^. All the materials were sterilized before using in the experiments. The classification of soil is clay and mixing with silt. The water content and the organic content were 28% and 8%, respectively which was measured by following the Japanese Geotechnical Society Standard (JGS). By saturated the soil with artificial sea water (saline water) for 3 days and then air drying it for 14 days, the final water content of the saline soil was around 5–6%. Three replicas were created, and each soil specimen was prepared in 18 cm column with a diameter of 6 cm. The soil was filled with up to 15 cm. The acrylic columns were wrapped with a black tape to avoid possible effects of sunlight. There were 3 positions from the side to collect the soil samples. The bacteria used in experiment 1 is listed in Table 1. The bacteria (in liquid form, 20 ml) were mixed in the same amount of saline soil (600 g) to observe the effectiveness of each bacterium as the potential of bioremediation. Two collective bacteria called *CM 1(Lactic acid bacteria, fermentive bacteria)* and *CM2 (Lactic acid bacteria, fermentive bacteria, Photosynthetic bacteria)* were prepared at laboratory as effective microorganism to be used in the experiments. The other individual bacteria such as *Lactic acid bacteria* and *Photosynthetic bacteria* were also used. The reasons behind choosing these microorganisms were the salt tolerance ability of these bacteria and also, they are not pathogenic. All the bacteria which were used in this research are easily available in the natural environment and can live and grow with natural temperature.

**Table 1:** Different cases for the bioremediation in experiment 1 in the laboratory.

Case	Sample	Bacteria used
A	Saline soil	Blank
B	Saline soil	*Lactic Acid Bacteria *
C	Saline soil	*Photosynthetic Bacteria*
D	Saline soil	CM1 (Lactic acid bacteria, fermenting bacteria)
E	Saline soil	CM 2 (Lactic acid bacteria, photosynthetic bacteria)

Figure 8 illustrates the schematic diagram of Experiments. In Experiment 1, diverse bacteria were tried to see the effectiveness of the bioremediation. In experiment 2, a 3 cm thick FGW layer was placed in the middle and at the bottom, respectively. Top, bottom, top1 and middle 1 are the sample collection points. In experiment 3, FWG was mixed thoroughly with the saline soil. As the collective microorganism CM2 showed the best performance in experiment 1, it was used for Experiment 2 and 3.

The electrical conductivity (EC) and pH were measured by using portable pH meter and EC meter (Horiba D-54). The Japanese Geotechnical Society (JGS) standard for pH and EC measurement of soil was followed. The salinity of the soil was measured once a week by using EC measurement^15,17^. All laboratory tests were carried out in a fixed ambient temperature of 25 °C to avoid the effect of temperature^8,29^. The water content was fixed to be around 20% by providing water once a week which was also monitored during the experiment by conducting the water content test by following Japanese geotechnical society (JGS) method^30^.

#### Statistical analysis

For statistically significant testing, p (probability value) less than or equal to 0.05 was used. Statistical analysis was carried out using Microsoft Excel 2019 (Microsoft Corporation, USA).
